# Identification of pyroptosis-related lncRNA signature and AC005253.1 as a pyroptosis-related oncogene in prostate cancer

**DOI:** 10.3389/fonc.2022.991165

**Published:** 2022-09-29

**Authors:** JiangFan Yu, Rui Tang, JinYu Li

**Affiliations:** ^1^ Department of Dermatology, Second Xiangya Hospital, Central South University, Changsha, China; ^2^ Department of Rheumatology and Immunology, Second Xiangya Hospital, Central South University, Changsha, China; ^3^ Department of General Surgery, Second Xiangya Hospital, Central South University, Changsha, China

**Keywords:** prostate cancer, lncRNA, pyroptosis, immunity, tumor biomarkers, machine learning

## Abstract

**Background:**

Pyroptosis and prostate cancer (PCa) are closely related. The role of pyroptosis-related long non-coding RNAs (lncRNAs) (PRLs) in PCa remains elusive. This study aimed to explore the relationship between PRL and PCa prognosis.

**Methods:**

Gene expression and clinical signatures were obtained from The Cancer Genome Atlas and Gene Expression Omnibus databases. A PRL risk prediction model was established by survival random forest analysis and least absolute shrinkage and selection operator regression. Functional enrichment, immune status, immune checkpoints, genetic mutations, and drug susceptibility analyses related to risk scores were performed by the single-sample gene set enrichment analysis, gene set variation analysis, and copy number variation analysis. PRL expression was verified in PCa cells. Cell Counting Kit-8, 5-ethynyl-2′-deoxyuridine, wound healing, transwell, and Western blotting assay were used to detect the proliferation, migration, invasion, and pyroptosis of PCa cells, respectively.

**Results:**

Prognostic features based on six PRL (AC129507.1, AC005253.1, AC127502.2, AC068580.3, LIMD1-AS1, and LINC01852) were constructed, and patients in the high-score group had a worse prognosis than those in the low-score group. This feature was determined to be independent by Cox regression analysis, and the area under the curve of the 1-, 3-, and 5-year receiver operating characteristic curves in the testing cohort was 1, 0.93, and 0.92, respectively. Moreover, the external cohort validation confirmed the robustness of the PRL risk prediction model. There was a clear distinction between the immune status of the two groups. The expression of multiple immune checkpoints was also reduced in the high-score group. Gene mutation proportion in the high-score group increased, and the sensitivity to drugs increased significantly. Six PRLs were upregulated in PCa cells. Silencing of AC005253.1 inhibited cell proliferation, migration, and invasion in DU145 and PC-3 cells. Moreover, silencing of AC005253.1 promoted pyroptosis and inflammasome AIM2 expression.

**Conclusions:**

Overall, we constructed a prognostic model of PCa with six PRLs and identified their expression in PCa cells. The experimental verification showed that AC005253.1 could affect the proliferation, migration, and invasion abilities of PCa cells. Meanwhile, AC005253.1 may play an important role in PCa by affecting pyroptosis through the AIM2 inflammasome. This result requires further research for verification.

## Introduction

Prostate cancer (PCa) is the second most common cancer in the male population worldwide and is one of the top five causes of cancer-related death (1). Epidemiological examinations and clinical studies have found that the incidence of PCa is still rising (2). The symptoms of patients with early-stage PCa are non-specific, so PCa is often found in the middle and late stages, and surgical treatment at this time has a poor prognosis and a low survival rate (3). With several important recent discoveries in immune mechanisms and advanced molecular diagnostic platforms, immunotherapy is emerging as a viable option for PCa, especially castration-resistant PCa, to stimulate antitumor immunity (4, 5). Different patient responses to the same immunotherapy have been observed in patients with different types and stages of cancer (6). Moreover, the patient response depends on multiple factors, including intratumoral heterogeneity and prior treatment history, suggesting that the need for individualized and combined therapy is an important direction for future successful immunotherapy (7, 8). Diagnostic, prognostic, and predictive biomarkers enable patient-specific management of PCa (9). Specific biomarkers to facilitate the clinical selection of immunotherapy patients include programmed death ligand 1 (PD-L1) and prostatic acid phosphatase (PAP), but these approaches are limited by tumor heterogeneity or small percentage populations (10, 11). Therefore, there is an urgent need to identify new and effective biomarkers to establish a prognostic model of PCa.

Pyroptosis, distinct from apoptosis, is a type of programmed cell death induced by the inflammasome and carried out by gasdermin proteins (12). It is characterized by cell rupture and many pro-inflammatory factors being discharged (13, 14). Pyroptosis affects tumor cell invasion, multiplication, and migration, affecting cancer prognosis (15). The association between pyroptosis and cancer is highly intricate because as a way of cell death, pyroptosis could inhibit cancer occurrence and development. Meanwhile, the release of inflammatory mediators and various signaling pathways in pyroptosis is associated with tumorigenesis and resistance to chemotherapy (16, 17). Due to the close association between pyroptosis and cancer progress and prognosis, various prognostic biomarker studies based on pyroptosis genes have been identified and used to construct gene signatures with predictive power. For example, risk signatures based on five pyroptosis-related genes (PRGs) were biomarkers to predict the immunological condition and the outcome of lung adenocarcinoma (18). Furthermore, the expression of four PRG features strongly predicted a breast cancer patient’s prognosis (19). Nonetheless, the predictive merit of pyroptosis gene signatures in the prostate has not been completely clarified.

Long non-coding RNA (lncRNA) does not have the protein-coding capacity, and its length exceeds 200 nucleotides (20). LncRNA has been shown to play key functions in a variety of biological and disease processes, including cancer (21, 22). Growing evidence supports the involvement of lncRNA in PCa progression, including cell proliferation, apoptosis, metastasis, and invasion (23, 24). For example, overexpression of lncRNA PCAT14 inhibits the invasion of PCa cells and correlates with a good prognosis of PCa, which can be a diagnostic marker (25, 26). However, the role of pyroptosis-related lncRNAs (PRLs) in PCa still requires further exploration. Thus, exploring lncRNA biomarkers associated with pyroptosis in PCa is of clinical importance.

Machine learning is a branch of artificial intelligence that has been rapidly developed and applied in the field of medicine (27). Predictive models of diseases based on machine learning have been extensively mined (28). For example, Wu et al. used an ensemble of machine learning to develop a novel pyroptosis scoring system based on six lncRNAs to predict the prognosis of patients with low-grade glioma (29). In the present study, we used a machine learning approach to construct a PRL risk signature for PCa prognosis. Then, we investigated the associations of risk score models and clinical features, immune microenvironment, immune checkpoints, genetic mutations, and drug sensitivity to provide potential diagnostic and prognostic biomarkers for PCa. This study may help to understand the effect of PRL on PCa prognosis.

## Materials and methods

### Data sets and pretreatments

The Cancer Genome Atlas (TCGA)-PRAD (Prostate Adenocarcinoma) dataset was downloaded from UCSC Xena (https://xenabrowser.net/). The data processing was performed to obtain FPKM data directly from TCGA and convert it into TPM value, normalized by log_2_ (TPM+1). The GSE116918 (GPL25318) dataset was from Gene Expression Omnibus (GEO) (https://www.ncbi.nlm.nih.gov/gds/). For this validation cohort, 248 localized/locally advanced PCa patients commencing radical radiotherapy (with androgen deprivation therapy (ADT)) were included. The Affymetrix platform was utilized to generate raw data from the GSE116918 (GPL25318) dataset. The robust multi-chip averaging (RMA) algorithm was used to achieve background correction and normalization.

### Construction of a machine learning prognostic model for pyroptosis-related lncRNA

Forty-four pyroptosis genes (AIM2, APIP, CASP1, CASP3, CASP4, CASP5, CASP6, CASP8, CASP9, DHX9, DDX58, ELANE, GSDMA, GSDMB, GSDMC, GSDMD, GSDME, IFI16, IL18, IL1B, MAPK8, MAPK9, NAIP, NFKB1, NFKB2, NLRC3, NLRC4, NLRP1, NLRP12, NLRP2, NLRP3, NLRP6, NLRP7, NLRP9, NOD1, NOD2, PJVK, PLCG1, PRKACA, SCAF11, TIRAP, TNF, GPX4, and IL6) were obtained from the literature (30–37). The names and abbreviations of the 44 pyroptosis-related genes are shown in **Table 1**. Gene set variation analysis (GSVA) was used to derive the pyroptosis score. LncRNA was then used to do correlation analysis with pyroptosis score and select the genes with |correlation coefficient| > 0.3 and p < 0.05. These genes were subjected to univariate analysis, p < 0.05, and the single-factor meaningful genes were selected. Then, survival random forest was used to perform dimensionality reduction analysis to screen important genes. The screening criteria were rel. importance > 0.2. Next, important gene variables were screened out, and these important gene variables were used for the least absolute shrinkage and selection operator (Lasso) regression to construct a risk score model. The risk score was the sum of gene expression values × coefficients. The flowchart of this study is presented in **Figure S1**.

**Table 1:** Pyroptosis gene members.

Genes	Full names
AIM2	Absent in melanoma 2
CASP1	Cysteine-aspartic acid protease-1
CASP3	Cysteine-aspartic acid protease-3
CASP4	Cysteine-aspartic acid protease-4
CASP5	Cysteine-aspartic acid protease-5
CASP6	Cysteine-aspartic acid protease-6
CASP8	Cysteine-aspartic acid protease-8
CASP9	Cysteine-aspartic acid protease-9
ELANE	Elastase, neutrophil expressed
GPX4	Glutathione peroxidase 4
GSDMA	Gasdermin A
GSDMB	Gasdermin B
GSDMC	Gasdermin C
GSDMD	Gasdermin D
GSDME	Gasdermin E
IL18	Interleukin 18
IL1B	Interleukin 1 beta
IL6	Interleukin 6
NLRC4	NLR family CARD domain containing 4
NLRP1	NLR family pyrin domain containing 1
NLRP2	NLR family pyrin domain containing 2
NLRP3	NLR family pyrin domain containing 3
NLRP6	NLR family pyrin domain containing 6
NLRP7	NLR family pyrin domain containing 7
NOD1	Nucleotide-binding oligomerization domain containing 1
NOD2	Nucleotide-binding oligomerization domain containing 2
PJVK	Pejvakin/deafness, autosomal recessive 59
PLCG1	Phospholipase C gamma 1
PRKACA	Protein kinase cAMP-activated catalytic subunit alpha
SCAF11	SR-related CTD-associated factor 11
TIRAP	TIR domain-containing adaptor protein
TNF	Tumor necrosis factor
APIP	Apoptotic protease activating factor 1-interacting protein
DHX9	DExH-box helicase 9
NLRP9	NLR family pyrin domain containing 9
NAIP	NLR family apoptosis inhibitory protein
IFI16	Interferon gamma inducible protein 16
NFKB1	Nuclear factor kappa B subunit 1
DDX58	Retinoic acid-inducible gene I
MAPK8	Mitogen-activated protein kinase 8
NLRC3	NLR family CARD domain containing 3
NLRP12	NLR family pyrin domain containing 12
MAPK9	Mitogen-activated protein kinase 9
NFKB2	Nuclear factor kappa B subunit 2

### Pathway and immune-infiltration evaluation

The single-sample gene set enrichment analysis (ssGSEA) algorithm was used to quantify the abundance of 28 immune cells (38) in PRAD and to compare immune infiltration with prognostic scores. Stromal score, Immune Score, ESTIMATE Score, and Tumor Purity were assessed with the ESTIMATE package, and the relationship between these scores and prognostic scores was compared. The GSVA package was used for GSVA analysis of Gene Ontology (GO) and the Kyoto Encyclopedia of Genes and Genomes (KEGG). The correlation analysis was performed with prognostic and functional enrichment pathway scores. Correlation analysis was performed with prognostic scores and all genes, and then gene set enrichment analysis (GSEA) was performed by the clusterProfiler package.

### Gene mutation and copy number variation analysis

The gene mutations of the two groups were compared, and the gene mutation patterns were checked. The somatic mutations and somatic copy number variation (CNV) profiles were gathered from the TCGA-PRAD datasets. The Genomic Identification of Significant Targets in Cancer (GISTIC) analysis was performed to evaluate the genomic features. The CNV landscape based on ITGA5 levels and the copy number gains or losses at the amplified or deleted peaks were assessed by GISTIC 2.0 analysis (https://gatk.broadinstitute.org).

### Drug prediction

Information on the sensitivity of tumor cell lines to potential drugs was downloaded from Cancer Therapeutics Response Portal 2 (CTRP v2) and Profiling Relative Inhibition Simultaneously in Mixtures (PRISM). The lower the area under the curve (AUC) of the cell line, the higher the sensitivity to the potential drug. Expression of cancer cell lines was downloaded from Cancer Cell Line Encyclopedia (CCLE). Predictions were made using the R package of pRRophetic.

### Cell culture and quantitative reverse transcription PCR

DU145 cells (BLUEFBIO, Shanghai, China) were grown in Dulbecco’s modified Eagle’s medium (DMEM) (complemented with 10% fetal bovine serum (FBS) and 1% penicillin–streptomycin (P/S)). PC-3 cells (Pricella, Wuhan, China) were grown in Ham’s F-12K media (complemented with 10% FBS and 1% P/S). RWPE1 cells (Abiowell, Changsha, China) were cultured in keratinocyte serum-free medium (K-SFM) (complemented with 50 mg/ml of bovine pituitary extract, 5 ng/ml of epidermal growth factor (EGF), and 1% P/S).

Total RNA was obtained using TRIzol reagent (Invitrogen, Carlsbad, CA, USA) from RWPE1, PC-3, and DU145 cells. RNA was reverse transcribed into cDNA using an mRNA reverse transcription kit (CW2569, CWBIO, Beijing, China). LncRNA expression was detected using the SYBR method (CW2601, CWBIO, China) and quantitative reverse transcription PCR (RT-qPCR) analysis with GAPDH as an internal reference. The primer sequences are shown in **Table 2**. The relative expression levels of genes were investigated by 2^−ΔΔCt^.

**Table 2:** Primer sequences.

Genes	Sequences (5′–3′)
GAPDH	F: ACAGCCTCAAGATCATCAGC
	R: GGTCATGAGTCCTTCCACGAT
AC005253.1	F: AAGCCTTCCCTGATTACTGC
	R: CATGGTCAAACAGCCTACCTC
AC068580.3	F: CACAGCCAAAACCAAACTCCT
	R: TGGGTTGCCATTCACTGACT
AC127502.2	F: CTTCTGAATCTTTCCGGCGAAC
	R: GCGAACAACCTTCCTTGCAAA
AC129507.1	F: CTTCACTCGCACGGAGCAAC
	R: CCTCCTTGCTGCCGAGTCA
LIMD1-AS1	F: TTTGATGCCGCTTTGCTCAC
	R: TGCCACTTTTCCAGGTGTGT
LINC01852	F: GCCGGAGAACGAATGTGATG
	R: TCTTTTTGTTTACCGGAGTTCCA

### Cell transfection

The small interference RNA (siRNA) specifically targeting AC005253.1 (si-AC005253.1-1: 5′-CCGCAAGAAGAAGUGUGGUCATT-3′, 5′-UGACCACACUUCUUCUUGCGGTT-3′, si-AC005253.1-2: 5′-GCGUCCCAAGAAGAAGGUCAATT-3′, 5′-UUGACCUUCUUCUUGGGACGCTT-3′ and si-AC005253.1 -3: 5′-GCGUCUGAUAUUUGCCGGCAATT-3′, 5′-UUGCCGGCAAAUAUCAGACGCTT-3′) and the corresponding negative controls (si-NC: 5′-UUCUCCGAACGUGUCACGUTT-3′, 5′-ACGUGACACGUUCGGAGAATT-3′) were obtained from Sangon Biotech (Shanghai, China). According to the manufacturer’s protocol, cells were transfected with Lipofectamine 3000 reagent (Thermo Fisher, Waltham, MA, USA) (39).

### Cell counting kit-8 assay

Cells were digested, counted, and seeded in a 96-well plate (5 × 10^3^ cells/well, 100 μl). After adherent cell culture, 10 μl of Cell Counting Kit-8 (CCK-8) solution (NU679, Dojindo, Tokyo, Japan) was added. The cells were incubated at 37°C with 5% CO_2_ for 4 h, and the optical density (OD) value at 450 nm was measured with a Bio-Tek microplate reader (MB-530, HEALES) (40).

### 5-Ethynyl-2′-deoxyuridine assay

The 5-ethynyl-2′-deoxyuridine (EDU) assay kit (Guangzhou RiboBio, Guangzhou, China) was used to monitor cell proliferation. The cells were inoculated into 96-well plates (1 × 10^4^/well) until 80% confluence. Each well was added with 100 μl of EDU solution and incubated for 2 h. The cells were incubated with 4% paraformaldehyde at room temperature for 30 min. Then, the cells were treated with 100 μl of 1× Apollo^®^ staining reaction solution for 30 min. Next, 100 μl of Hoechst 33342 reaction solution was added to each well and incubated for 30 min. A microscope (DSZ2000X, Beijing Cnmicro Instrument Co., Ltd., Beijing, China) was used to observe and take pictures.

### Wound healing assay

Cells (1 × 10^5^/well) were plated in 6-well plates until they achieved about 90% confluence. A 1-ml pipette tip was used to create scratch wounds, and photographs of the wounds (time 0 h) were immediately taken. Then, the cells were cultured in a serum-free medium. After incubation with 5% CO_2_ at 37°C for 48 h, photographs were taken again.

### Transwell assay

The invasion ability of cells was evaluated using the transwell assay. Transwell chambers (3428, Corning, New York, NY, USA) were pre-cooled overnight at 4°C one day in advance. Then, 100 μl of Matrigel dilute in serum-free medium was added to each well. The transfected cells were suspended in a serum-free medium and added to the transwell chamber; 500 μl of 10% fetal bovine serum complete medium was placed into the lower chamber. The cells were incubated at 37°C for 48 h. The upper chamber was removed and washed three times with phosphate-buffered saline (PBS), and the cells in the upper layer of the membrane were wiped off with a cotton ball. Cells were fixed with 4% paraformaldehyde for 20 min and stained with 0.1% crystal violet solution for 5 min. Cells were observed under an inverted microscope, and three fields of view were taken. The number of invasive cells was recorded.

### Western blotting

Total protein was extracted from the cells by radioimmunoprecipitation assay (RIPA) lysate (AWB0136, Abiowell, China). Then, the protein was transferred to the polyvinylidene fluoride membrane after 10% sodium dodecyl sulfate–polyacrylamide gel electrophoresis (SDS-PAGE) treatment. The membrane was sealed with 5% skim milk (AWB0004, Abiowell) at room temperature for 2 h. AIM2 (1:1,500, 20590-1-AP, proteintech, Chicago, IL, USA), NLRC4 (1:1,000, ab201792, abcam, Cambridge, UK), NLRP3 (1:1,000, 19771-1-AP, proteintech), GSDMD-N (1:1,000, ab215203, abcam), ASC (1:2,000, 10500-1-AP, proteintech), caspase-1 (1:1,000, ab179515, abcam), IL-18 (1:8,000, 10663-1-AP, proteintech), IL-1β (1:1,000, 16806-1-AP, proteintech), and β-actin (1:5,000, 66009-1-Ig, proteintech) were incubated with the membrane at 4°C overnight. Then, the corresponding secondary antibodies were incubated with the membrane at room temperature for 2 h. The membrane was incubated with SuperECL Plus (AWB0005, abiowell), and then the protein bands were visualized by a chemiluminescence imaging system (ChemiScope 6100, Clinx, Shanghai, China).

### Statistical analysis

The data were mainly visualized using the R package ggplot2. The Shapiro–Wilk normality test was used to evaluate for normality of variables. For normally distributed variables, significant quantitative differences were determined by two-tailed t-tests or one-way ANOVA. For non-normally distributed variables, significant quantitative differences were determined by the Wilcoxon test or the Kruskal–Wallis test. The Benjamini–Hochberg method was used, which converts p-values to false discovery rate (FDR) to identify significant genes. The log-rank test was used to determine the statistical differences in each dataset. The Survminer R package was used to generate survival curves. Receiver operating characteristic (ROC) curves were drawn using the pROC package. All heatmaps were generated based on pheatmap. All statistical analyses were performed in R (https://www.r-project.org/). p < 0.05 was considered statistically significant.

## Results

### Construction of pyroptosis-related lncRNA signatures

**Figure 1:**
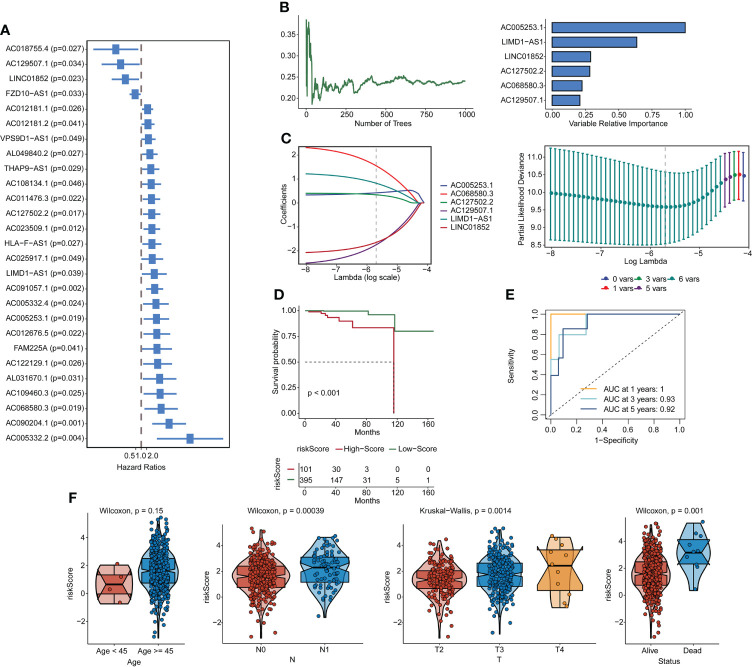
Construction of pyroptosis-related lncRNA signatures. **(A)** Correlation gene screens for genes of univariate significance. **(B, C)** Six PRL signatures were constructed through a random forest and Lasso analysis. **(D)** Survival curves. **(E)** ROC curves. **(F)** Clinical feature scores. lncRNA, long non-coding RNA; PRL, pyroptosis-related lncRNA; Lasso, least absolute shrinkage and selection operator; ROC, receiver operating characteristic.

Forty-five pyroptosis genes were obtained from the literature, and the pyroptosis score was calculated by the GSVA method. The correlation between lncRNAs and pyroptosis scores was analyzed by Spearman’s correlation test. The lncRNAs with |correlation coefficient| > 0.3 and p < 0.05 were selected, and 553 lncRNAs were obtained. These lncRNAs were subjected to univariate analysis, and 27 lncRNAs (p < 0.05) were screened (**Figure 1A**). The single-factor meaningful genes were selected for survival random forest analysis, and six important lncRNAs were screened (**Figure 1B**, **Figure S2A**). These important gene variables were then used for Lasso analysis to build a risk scoring model (**Figure 1C**). The risk score was 0.4242 × AC005253.1 + 0.8249 × LIMD1-AS1 − 1.6456 × LINC01852 + 0.3408 × AC127502.2 + 1.5518 × AC068580.3 − 1.7451 × AC129507.1. With the median risk score as the cutoff value, PCa patients were categorized into high-score and low-score groups. PCa patients in the high-score group had poorer overall survival than those in the low-score group (**Figure 1D**). Furthermore, the AUC values of the 1-, 3-, and 5-year ROC curves were 1, 0.93, and 0.92, respectively (**Figure 1E**). The clinical characteristic score showed that the risk score of patients aged ≥45 was higher than that of patients aged <45, but there was no significant difference (p = 0.15). Patients with stage N1 had a higher risk score than patients with stage N0 (p = 0.00039). T4 stage patients had higher risk scores than T3 stage and T2 stage patients, and T3 stage patients had higher risk scores than T2 stage patients (p = 0.0014). Moreover, patients with the status alive had a lower risk score than patients with the status dead (p = 0.001) (**Figure 1F**). A publicly available dataset (GSE116918) was used to validate the reliability of the constructed risk scoring model. Consistent with the findings from the TCGA-PRAD cohort, survival analysis showed that patients in the high-score group had lower survival rates than those in the low-score group (**Figure S2B**). In addition, the AUC values of the 1-, 3-, and 5-year ROC curves were 0.7, 0.71, and 0.77, respectively (**Figure S2C**). To investigate whether the constructed risk scoring model was independent of clinicopathological parameters, univariate and multivariate Cox regression analyses were performed on age, T stage, N stage, and risk score. Risk score was the parameter independently predicting overall survival (**Figures S2D, E**). The predictive model could be considered an independent prognostic factor in PCa patients.

### Correlation of risk scores with pyroptosis genes and immune infiltration

**Figure 2:**
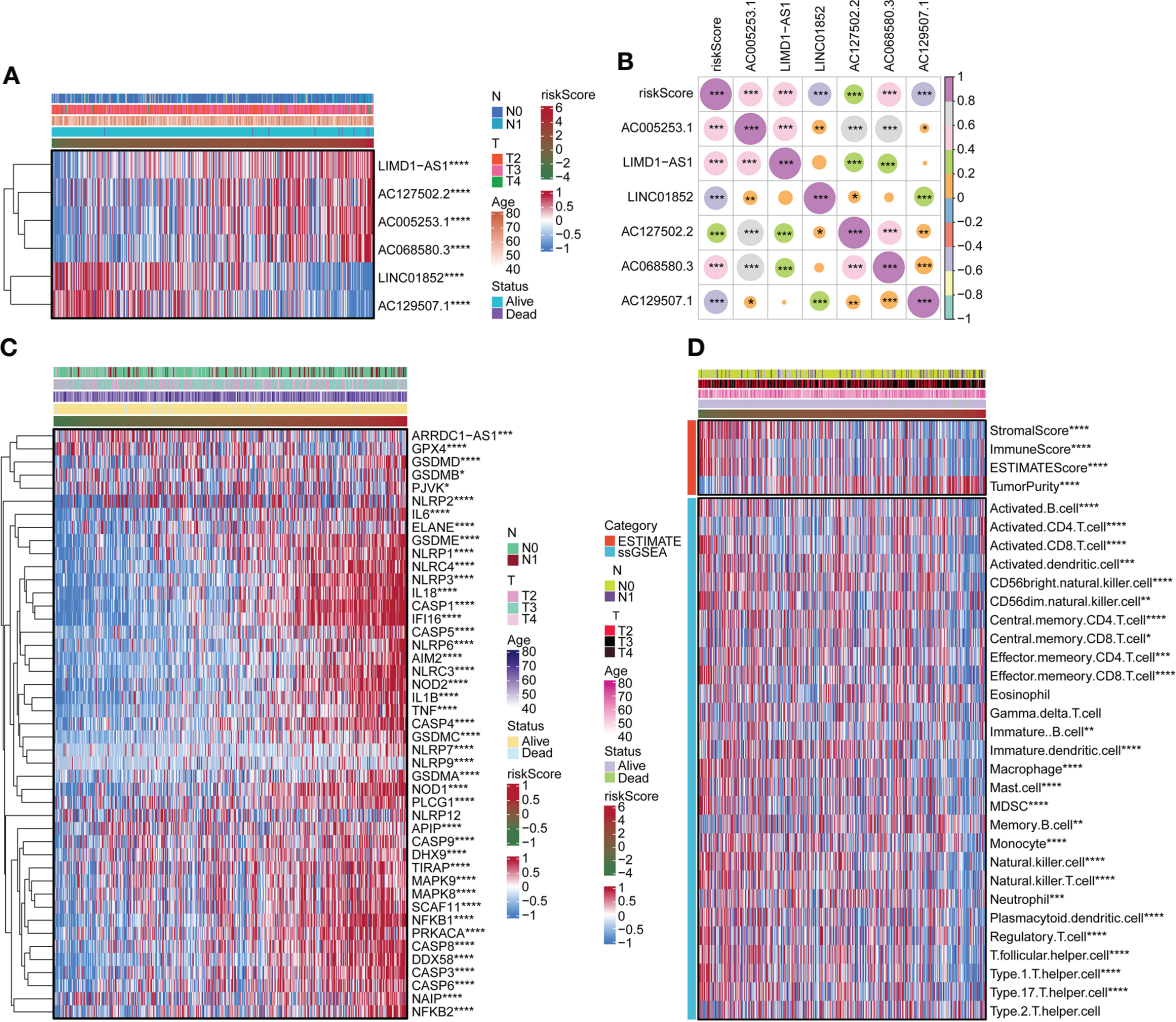
Correlation of risk scores with pyroptosis genes and immune infiltration. **(A)** The expression of LIMD1-AS1, AC127502.2, AC005253.1, AC068580.3, LINC01852, and AC129507.1. **(B)** Expression correlation plots of risk scores and model genes. **(C)** Heatmap of risk score associated with pyroptosis genes. **(D)** Heatmap of the relationship of the risk score to immune infiltration. *p < 0.05. **p < 0.01. ***p < 0.001 ****p < 0.0001.

We surveyed the relationship between model genes and risk scores. Risk scores were favorably associated with LIMD1-AS1, AC127502.2, AC005253.1, and AC06850.3 and negatively associated with LINC01852 and AC129507.1 (**Figures 2A, B**). Next, we constructed a heatmap of risk scores and pyroptosis gene correlations. The results showed that ARRDC1-AS1, GPX4, GSDMD, GSDME, and NLRP3 were substantially associated with the risk score (**Figure 2C**). We used the ESTIMATE package and ssGSEA algorithm to evaluate immune infiltration. Among them, Stromal score, Immune Score, and ESTIMATE Score were negatively associated with risk score, while Tumor Purity was positively associated with risk score (**Figure 2D**). B cell, T cell, Macrophage, and Natural killer cell were significantly associated with risk score (**Figure 2D**). For example, Activated B cell, Activated CD8 T cell, and Activated dendritic cell were negatively associated with risk scores. Activated CD4 T cell was positively associated with risk score.

### Immune checkpoint

**Figure 3:**
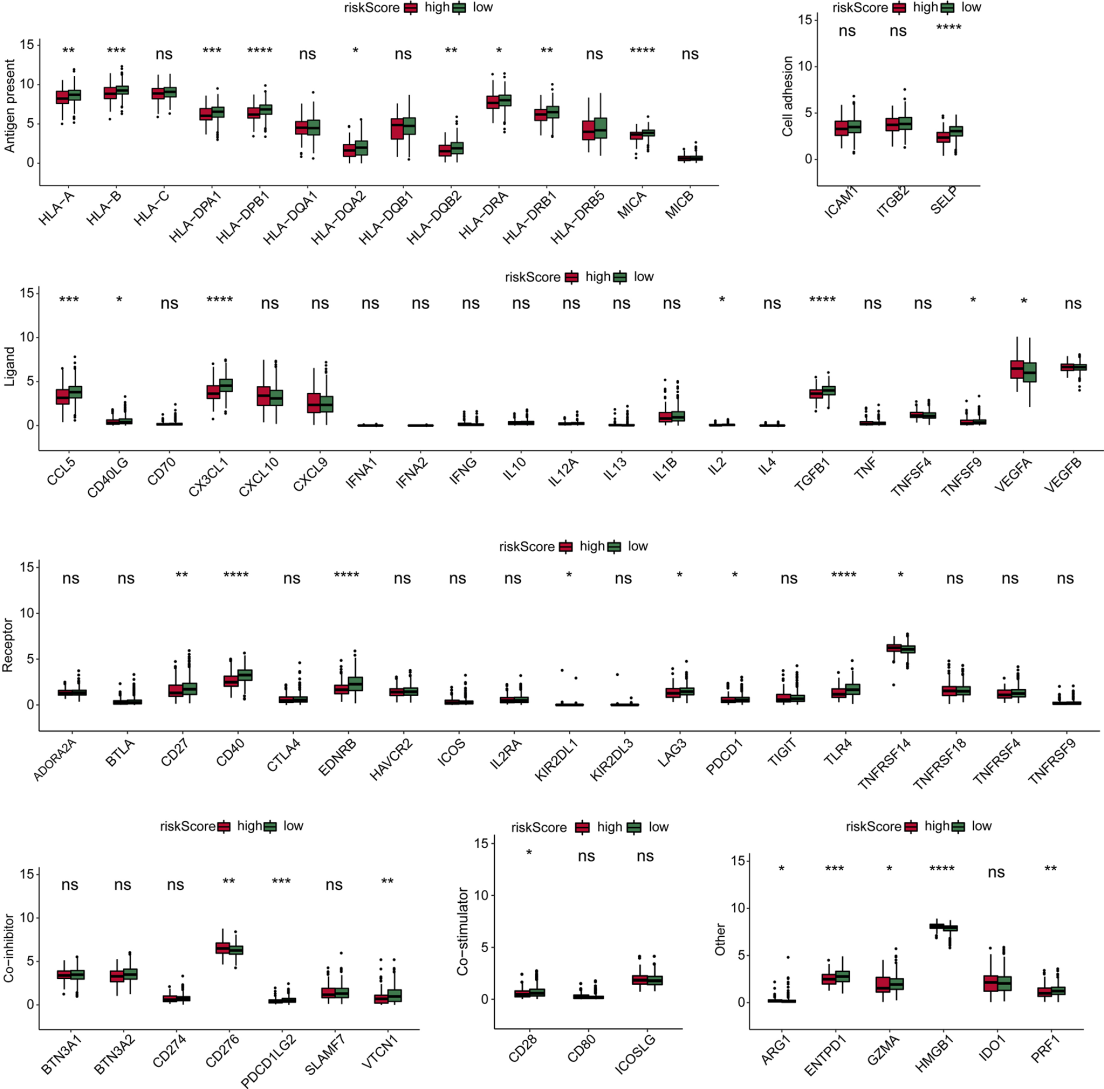
Immune checkpoint. Immune checkpoint molecule expression in low- and high-score groups. *p < 0.05. **p < 0.01. ***p < 0.001 ****p < 0.0001. ns, not significant.

We determined the expression of seven classes of immune checkpoint molecules in low- and high-risk-scoring populations. As shown in the Antigen present classification, HLA-A, HLA-B, HLA-DPA1, HLA-DPB1, HLA-DQB2, HLA-DRB1, and MICA were expressed at a low level in the high-score group. In Cell adhesion, SELP was expressed at a high level in the low-score group. In Ligand, CCL5, CX3CL1, and TGFB1 were expressed at a high level in the low-score group. In Receptor, CD27, CD40, EDNRB, and TLR4 were expressed at a high level in the low-score group. In Co-inhibitor, CD276 was expressed at a low level in the low-score group, and PDCD1LG2 and VTCN1 were expressed at a high level in the low-score group. In addition, HMGB1 was expressed at a low level in the low-score group, while ENTPD1 and PRF1 were expressed at a high level in the low-score group (**Figure 3**).

### Functional analysis of risk score

**Figure 4:**
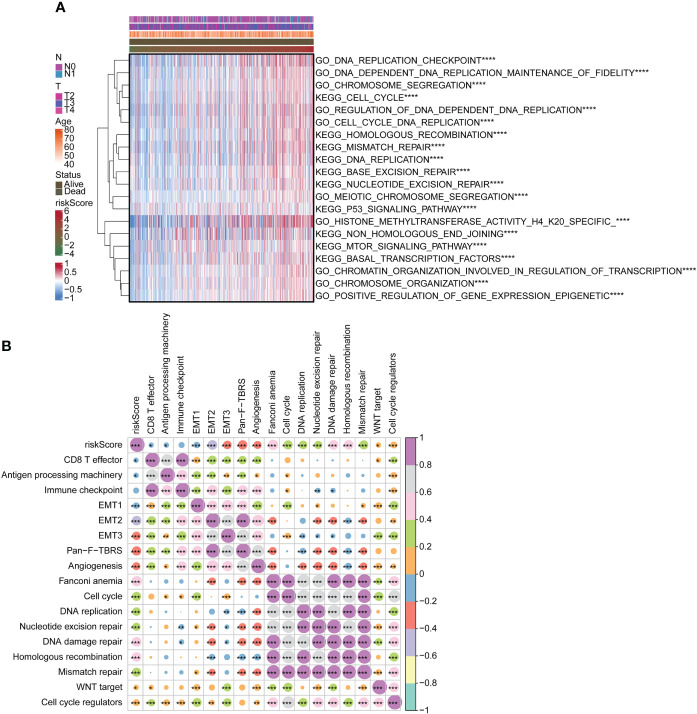
Functional analysis of risk score. **(A)** Heatmap for GO and KEGG analyses using GSVA package. **(B)** Correlation of risk score with functional enrichment pathways. GO, Gene Ontology; KEGG, Kyoto Encyclopedia of Genes and Genomes; GSVA, gene set variation analysis. *p < 0.05. **p < 0.01. ***p < 0.001 ****p < 0.0001.

We used the GSVA package for GO and KEGG enrichment analyses. Most samples were enriched for pathways closely related to tumorigenesis. Examples included DNA replication, cell cycle, and mTOR signaling pathway. These pathways were positively associated with risk scores (**Figure 4A**). Correlation analysis with risk score and functional enrichment pathway score was performed. In addition to the immune checkpoint, risk scores were significantly associated with 16 other pathways (**Figure 4B**). Risk scores were positively correlated with cell cycle, DNA replication, DNA damage repair, and WNT target while negatively correlated with CD8 T effector and antigen processing machinery. We conducted a relevant analysis with the risk score and all genes and then performed a GSEA with the clusterProfiler package (**Figure S3**). The Hippo signaling pathway was downregulated.

### Copy number variation and mutation analysis in high- and low-score groups

**Figure 5:**
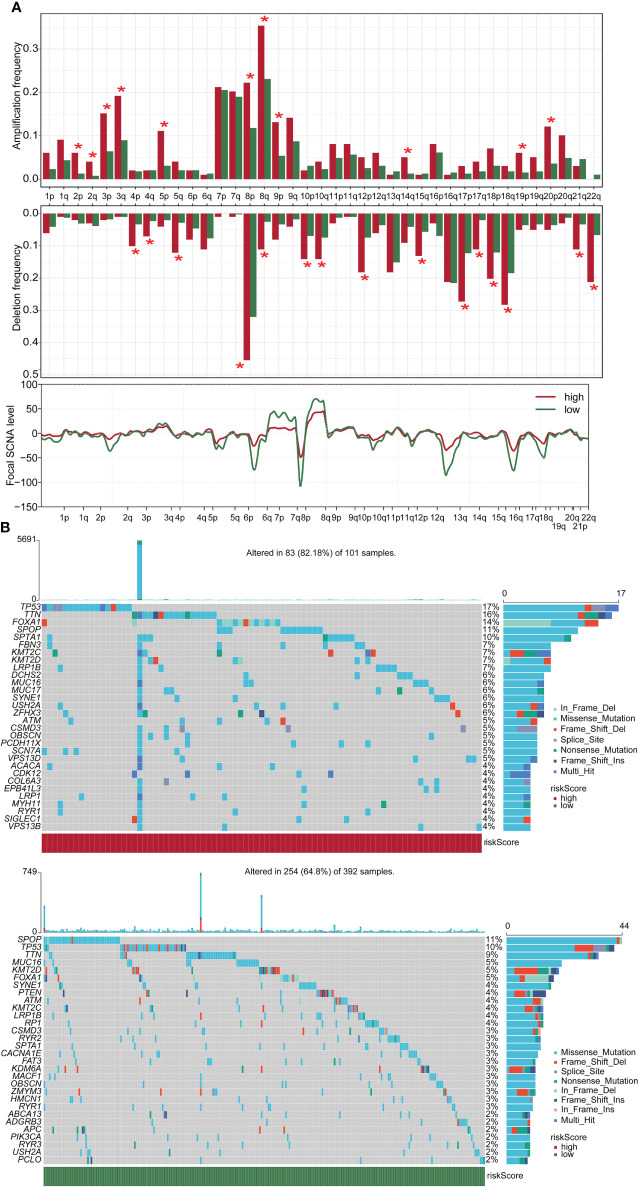
CNV and mutation analysis in high- and low-score groups. **(A)** CNV maps for groups with high and low score. **(B)** Top 30 gene mutation frequencies in two groups. CNV, copy number variation. *p < 0.05

In this study, we compared the gene mutation status of two groups. Amplification frequency was mainly concentrated in 2p, 2q, 3p, 3q, 5p, 8p, 8q, 9p, 14p, 19p, and 20p, while deletion frequency was mainly concentrated in 4p, 4q, 5q, 8p, 8q, 10p, 10q, 12p, 15q, 17p, 17q, 18p, 18q, 21q, and 22q. In addition, there was a significant difference between the high-risk and low-score groups at the focal somatic copy number alterations (SCNA) level (**Figure 5A**). A waterfall plot was used to visualize the mutation frequency and type of the top 30 genes with the highest gene mutation frequency. The results showed that in the high-risk group, the top five genes with the highest mutation frequency were TP53 (17%), TTN (16%), FOXA1 (14%), SPOP (11%), and SPTA1 (10%), while in the low-risk group the top five genes were SPOP (11%), TP53 (10%), TTN (9%), MUC16 (5%), and KMT2D (5%) (**Figure 5B**).

### Drug sensitivity analysis of risk scores in two groups

**Figure 6:**
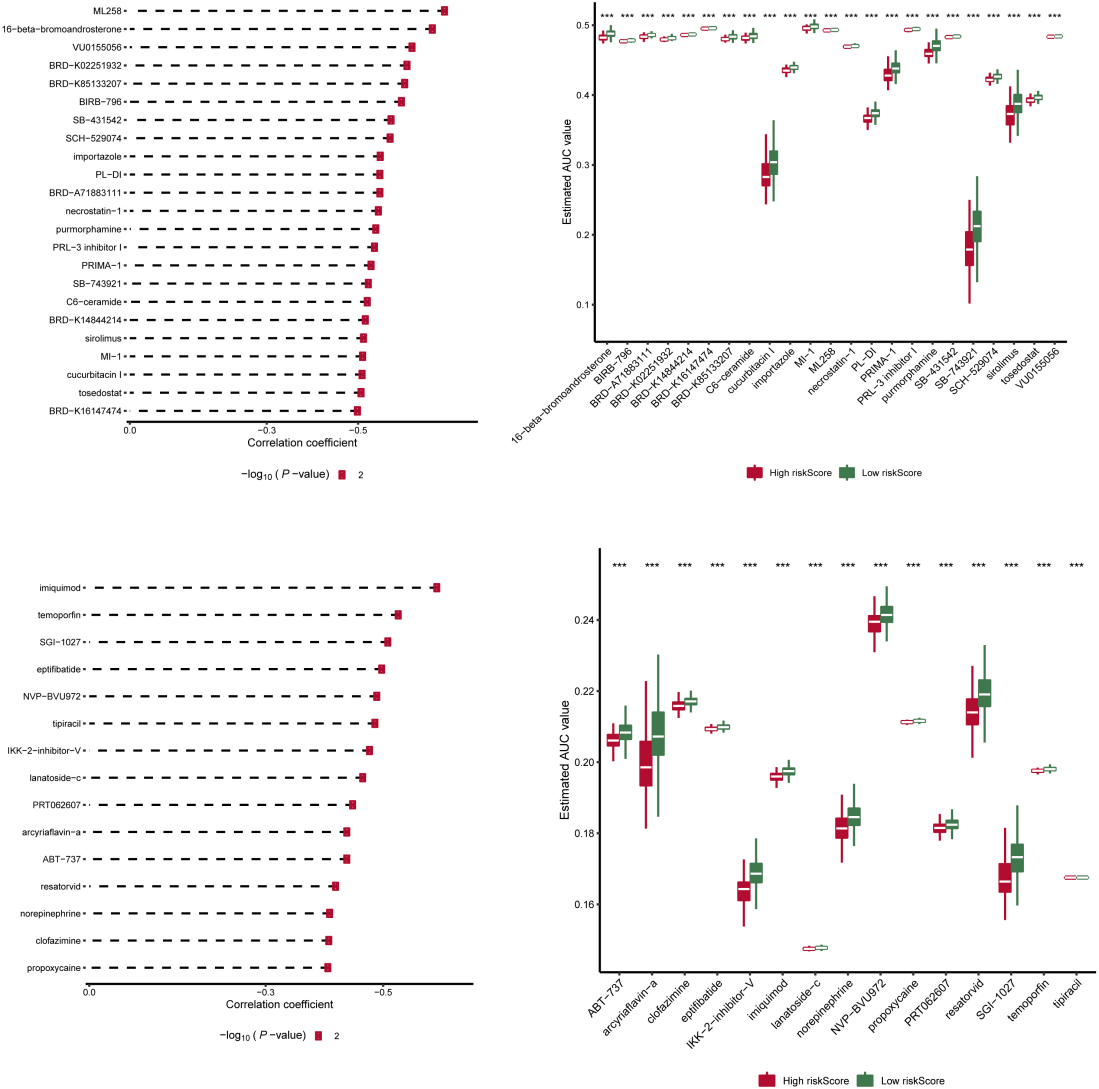
Drug sensitivity analysis of risk scores in two groups. Information on the sensitivity of tumor cell lines to potential drugs was downloaded from CTRP v2 and PRISM. Prediction of drug susceptibility in two groups using pRRophetic. The lower the AUC of the cell line, the higher the sensitivity to the potential drug. AUC, area under the curve. ***p < 0.001

We downloaded information on the susceptibility of tumor cell lines to potential drugs from the CTRP v2 and PRISM. The lower the AUC of the cell line, the higher the sensitivity to the potential drug. These data revealed that among anticancer drugs, including ML258, 16-beta-bromoandrosterone, VU0155056, BRD-K02251932, BRD-K85133207, imiquimod, temoporfin, SGI-1027, and eptifibatide, the sensitivity of patients in the high-score group to the drugs was significantly increased than in the low-score group (**Figure 6**).

### Risk score gene expression identification

**Figure 7:**
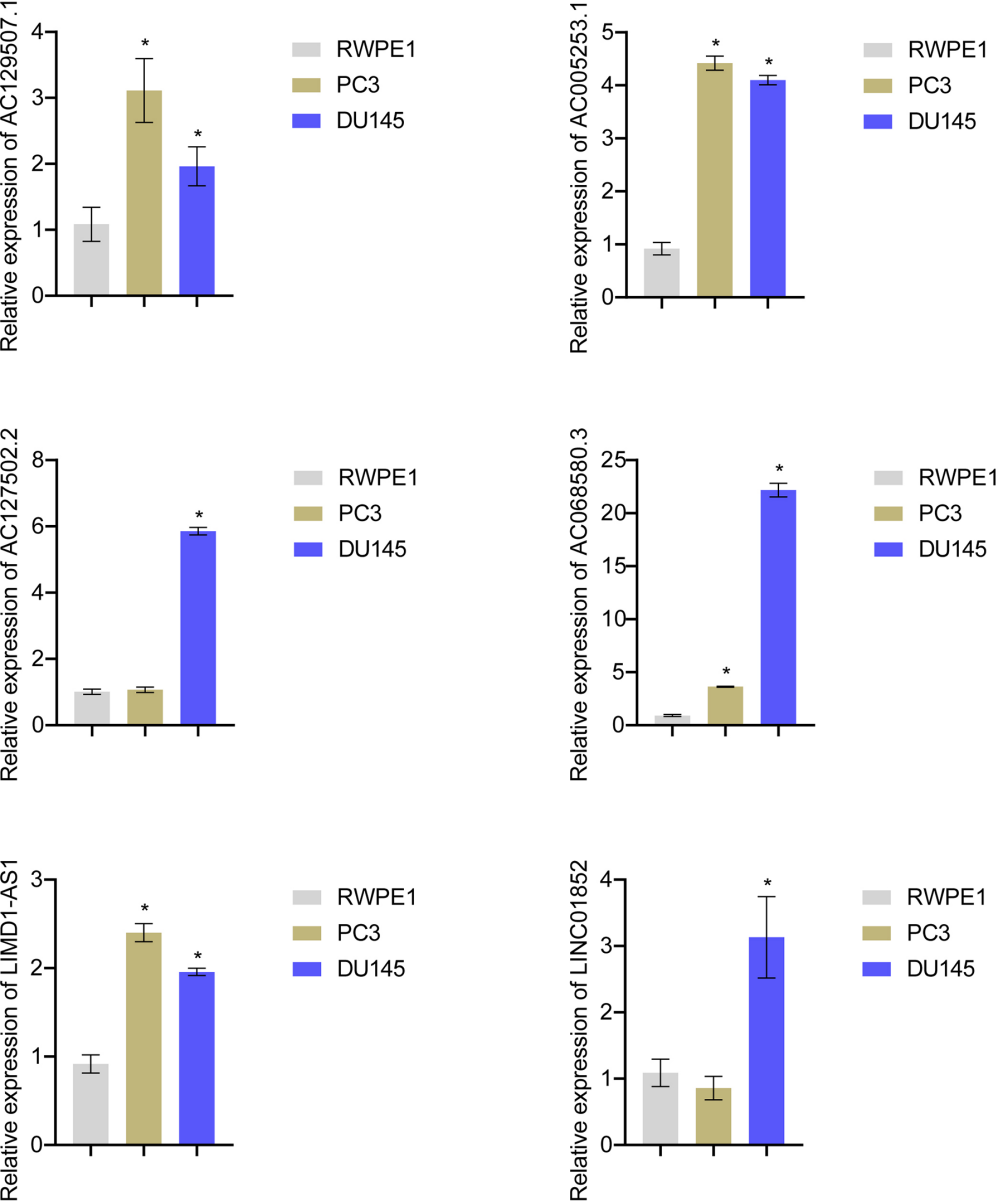
Risk score gene expression identification. RT-qPCR to detect the AC129507.1, AC005253.1, AC127502.2, AC068580.3, LIMD1-AS1, and LINC01852 expression in RWPE1, PC-3, and DU145 cell. *p < 0.05, *vs.* RWPE1.

Expression of AC129507.1, AC005253.1, AC127502.2, AC068580.3, LIMD1-AS1, and LINC01852 was verified by RT-qPCR in RWPE1 cells and PCa cell lines (PC-3 and DU145). The results showed that AC129507.1, AC005253.1, AC068580.3, and LIMD1-AS1 were upregulated in PC-3 and DU145 cells relative to RWPE1 cells. However, AC127502.2 and LINC01852 were only upregulated in DU145 cells (**Figure 7**).

### Silencing of AC005253.1 affected prostate cancer cell proliferation, migration, and invasion

**Figure 8:**
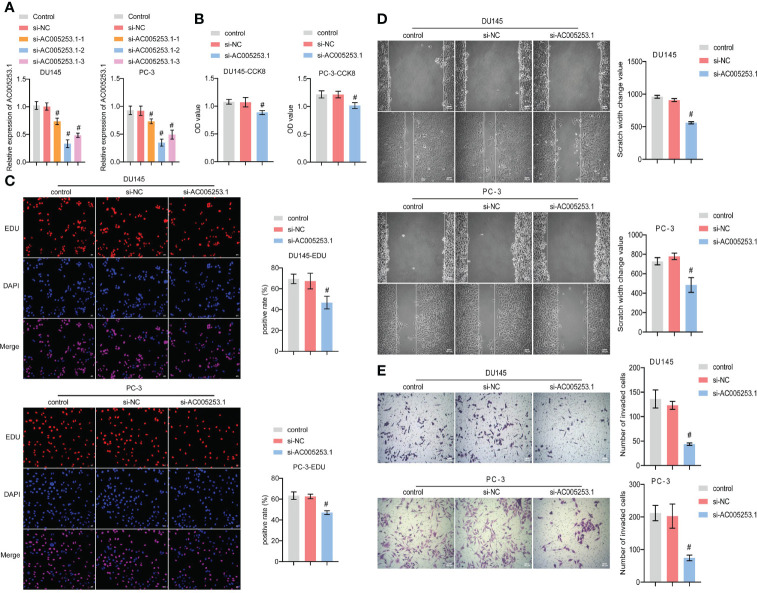
Silencing of AC005253.1 affected PCa cell proliferation, migration, and invasion. **(A)** RT-qPCR detection of AC005253.1 expression in PC-3 and DU145 cells. **(B)** CCK-8 assay was used to measure the cell viability in PC-3 and DU145 cells. **(C)** EDU assay results showed the effect of si-AC005253.1 on cell proliferation. **(D)** Wound healing assay was performed to detect the migration in PC-3 and DU145 cells. **(E)** Transwell assay was used to detect the invasion of PC-3 and DU145 cells. #p < 0.05, *vs.* si-NC group. PCa, prostate cancer; CCK-8, Cell Counting Kit-8.

To explore the role of AC005253.1 in the development of PCa, we transfected si-AC005253.1-1, si-AC005253.1-2, si-AC005253.1-3, and si-NC in PC-3 and DU145 cells. RT-qPCR results showed that si-AC005253.1-1, si-AC005253.1-2, and si-AC005253.1-3 could reduce the expression of AC005253.1 in PC-3 and DU145 cells, among which si-AC005253.1-2 had a best silencing effect (**Figure 8A**). Therefore, si-AC005253.1-2 was used as a follow-up experiment. Silencing of AC005253.1 decreased the cell viability and proliferation of PC-3 and DU145 cells (**Figures 8B, C**). The wound healing assay and transwell assay results showed that after silencing AC005253.1, the migration and invasion abilities of PC-3 and DU145 cells were reduced (**Figures 8D, E**).

### Silencing of AC005253.1 promoted pyroptosis of prostate cancer cells

**Figure 9:**
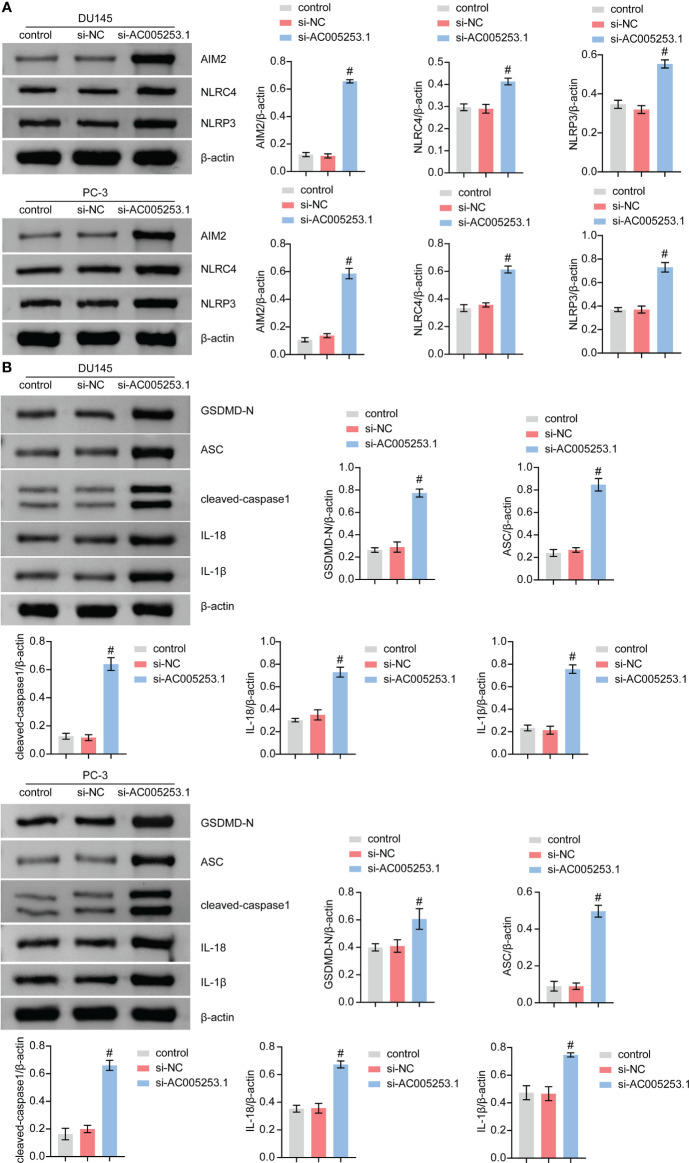
Silencing of AC005253.1 promoted pyroptosis of PCa cells. **(A)** AIM2, NLRC4, and NLRP3 levels were identified by Western blotting. **(B)** GSDMD-N, ASC, caspase-1, IL-18, and IL-1β proteins were identified by Western blotting in PC-3 and DU145 cells. #p < 0.05, *vs.* si-NC group. PCa, prostate cancer.

We further tested the effect of AC005253.1 on the pyroptosis of PC-3 and DU145 cells by Western blotting experiment. The results showed that after silencing AC005253.1, the expression of the inflammasomes (AIM2, NLRC4, and NLRP3) was altered (**Figure 9A**). Compared with the si-NC group, the expression of AIM2 was increased in the si-AC005253.1 group, and the difference was most obvious (**Figure 9A**). Furthermore, after silencing AC005253.1, the expressions of GSDMD-N, ASC, cleaved caspase-1, IL-18, and IL-1β proteins were increased in PC-3 and DU145 cells (**Figure 9B**). These results suggested that silencing of AC005253.1 promoted pyroptosis in PCa cells.

## Discussion

PCa is one of the most common tumors in men. Due to its heterogeneity and progressive nature, it remains incurable (41). Valid prognostic models based on specific biomarkers can accurately predict survival outcomes for the effective management of PCa patients (42). Pyroptosis-related lncRNA risk prediction models have been reported to be expected to assist in the treatment and management of various tumors (43, 44). Our study used a novel risk model of six PRLs (AC129507.1, AC005253.1, AC127502.2, AC068580.3, LIMD1-AS1, and LINC01852) developed using Lasso analysis. AC129507.1, AC005253.1, AC127502.2, AC068580.3, LIMD1-AS1, and LINC01852 were identified as PRLs for the first time. The results showed that the risk score model had the best ability to distinguish clinical characteristics between the high-risk and low-score groups significantly. In our proposed model, the AUC values of the ROC curves for 1-, 3-, and 5-year PCa were 1, 0.93, and 0.92, respectively. In addition, the 1-, 3-, and 5-year AUC values in the test set also had desirable results. Our risk model had excellent predictive power compared to other published pyroptosis-based prognostic models in PCa (45, 46). Different from the direct use of Lasso to build a prognostic model of eight pyroptosis-related genes in the study of Wang et al. (47), we used random forest dimensionality reduction and screening methods and further used Lasso analysis to build a predictive model. This analytical approach may help improve the predictive accuracy of the risk model signature.

It has been reported that pyroptosis is closely related to the tumor immune microenvironment. The release of inflammatory factors is caused by pyroptosis triggers powerful antitumor immunity (48). The infiltration of CD8+ T cells and natural killer cells in the pyroptosis-activated immune microenvironment can promote pyroptosis and form a positive feedback loop (49). Previous studies have shown that increased CD8+ T-cell infiltration is independently associated with improved survival after radical prostatectomy (50). Men with more CD4+ T cells in the prostate tumor environment have an increased risk of dying from PCa (51). B-cell activation is thought to be a driver of the PCa immune response and improves postoperative survival (52). Overall, the poor prognosis and outcome of PCa are closely related to pyroptosis-triggered immune cell infiltration, which is consistent with our results. Our results showed that the risk score was negatively correlated with activated B cells, CD8 T cells, and dendritic cells while positively correlated with activated CD4 T cells. Infiltration levels of B cells, CD8 T cells, and dendritic cells decreased with increasing risk scores, consistent with shorter survival times in patients with high scores.

We compared the expression of immune checkpoints in the high-risk and low-risk groups and found that some immune checkpoints such as HLA and MICA were expressed at a high level in the low-risk group. PCa downregulated the expression of the HLA-1 antigen processing machinery (APM) and had defects in the antigen presentation pathway (53). Low expression of MICA is associated with poorer overall survival in PCa and is associated with aggressiveness (54). This suggests that our signature could effectively identify the status of immune checkpoints in different PCa patients, providing new ideas for their treatment.

CNVs were regions of the genome with integer copy number changes, including amplifications and deletions of DNA sequences, that could drive cancer’s rapid adaptive evolution and progression (55). The CNV results uncovered significant differences in mutation status between the high-score and low-score groups. In the high-score group, the gene with the highest mutation frequency was TP53 (17%), while in the low-risk group, it was SPOP (11%). TP53 mutation was the most common genetic alteration that played a major role in the pathogenesis of PCa (56, 57). SPOP mutations were associated with improved overall survival, whereas TP53 mutations were associated with poorer survival in secondary metastatic hormone-sensitive PCa (58). These data implicated that the high-score group might have more tumorigenic gene mutations.

To better assess the risk model’s clinical feasibility, we analyzed information on the sensitivity of tumor cell lines to potential drugs. The results showed that the high-risk group cell lines were significantly less sensitive to drugs such as importazole and imiquimod. Importazole, a specific inhibitor that alters the interaction of KPNB1 with RanGTP, has a good inhibitory effect on PCa progression (59). Imiquimod (also known as a TLR7 agonist) inhibits the growth of mouse (TRAMP C2) and human PCa cells and can be used as an alternative therapy for locally generated PCa (60). Our findings suggest that the low-risk group is more likely to benefit from these drugs. Collectively, these findings may provide prospective treatment options for PCa patients.

Notably, RT-qPCR analysis confirmed high expression of AC129507.1, AC005253.1, AC127502.2, AC068580.3, LIMD1-AS1, and LINC01852 in PCa cell lines. AC129507.1, AC068580.3, and LIMD1-AS1 were thought to play important roles in different cancers, while AC005253.1, AC127502.2, and LINC01852 were identified for the first time. AC129507.1 was identified as an on-risk gene in risk models for prognosis patients with gastric adenocarcinoma (61). AC068580.3 was identified as an autophagy-related lncRNA as an indication of prognosis for colon adenocarcinoma (62). LIMD1-AS1 inhibited lung cancer progression by inhibiting cell multiplication and promoting apoptosis (63). Our study demonstrated that inhibiting the expression of AC005253.1 could inhibit cell viability, migration, and invasion. We further detected the expression of the inflammasome (AIM2, NLRC4, and NLRP3) and found that silencing of AC005253.1 could significantly increase the expression of the AIM2 inflammasome. Activation of the AIM2 inflammasome can promote pyroptosis (64). We also found that inhibition of AC005253.1 could promote pyroptosis in PCa cells. Therefore, we speculate that AC005253.1 may affect pyroptosis through the AIM2 inflammasome in PCa. In the present study, we report for the first time the relationship between AC005253.1 and pyroptosis in PCa.

In conclusion, we successfully established an efficient forecast PCa model based on six PRLs, including AC129507.1, AC005253.1, AC127502.2, AC068580.3, LIMD1-AS1, and LINC01852. This well-validated model built on these six PRLs will provide new insights into identifying PCa prognosis. Through *in vitro* experiments, we verified that silencing of AC005253.1 could inhibit the proliferation, migration, and invasion of PCa cells. In addition, silencing of AC005253.1 might promote pyroptosis by affecting the expression of AIM2 in PCa.

## Data availability statement

The original contributions presented in the study are included in the article/**Supplementary Material**. Further inquiries can be directed to the corresponding author.

## Author contributions

JL conceived the project. RT performed the experiments. JY analyzed the data. JY and JL wrote the paper. All authors contributed to the article and approved the submitted version.

## Funding

This research was funded by the Hunan Provincial Natural Science Foundation of China (2021JJ40868) and the National Natural Science Foundation of China (no. 82001738).

## Acknowledgments

We thank the authors who contributed to this study.

## Conflict of interest

The authors declare that the research was conducted in the absence of any commercial or financial relationships that could be construed as a potential conflict of interest.

## Publisher’s note

All claims expressed in this article are solely those of the authors and do not necessarily represent those of their affiliated organizations, or those of the publisher, the editors and the reviewers. Any product that may be evaluated in this article, or claim that may be made by its manufacturer, is not guaranteed or endorsed by the publisher.

## Supplementary material

Supplementary Figure S1 A flowchart of the study.

Supplementary Figure S2 Analysis of prognostic characteristics of risk score. (A) Univariate analysis of lncRNAs in TCGA. (B) Survival analysis of GSE116918. (C) ROC curves of GSE116918. (D, E) Univariate and multivariate Cox analysis.

Supplementary Figure S3 Enrichment analysis of risk score-related genes. After risk score correlation analysis with all genes, GO and KEGG functional enrichment analysis was performed by GSEA.
