# Supplementary figures and images for: Identification of pyroptosis-related lncRNA signature and AC005253.1 as a pyroptosis-related oncogene in prostate cancer
Source: Front Oncol. 2022 Sep 29;12:991165. doi: 10.3389/fonc.2022.991165 (PMC9556775; doi:10.3389/fonc.2022.991165)

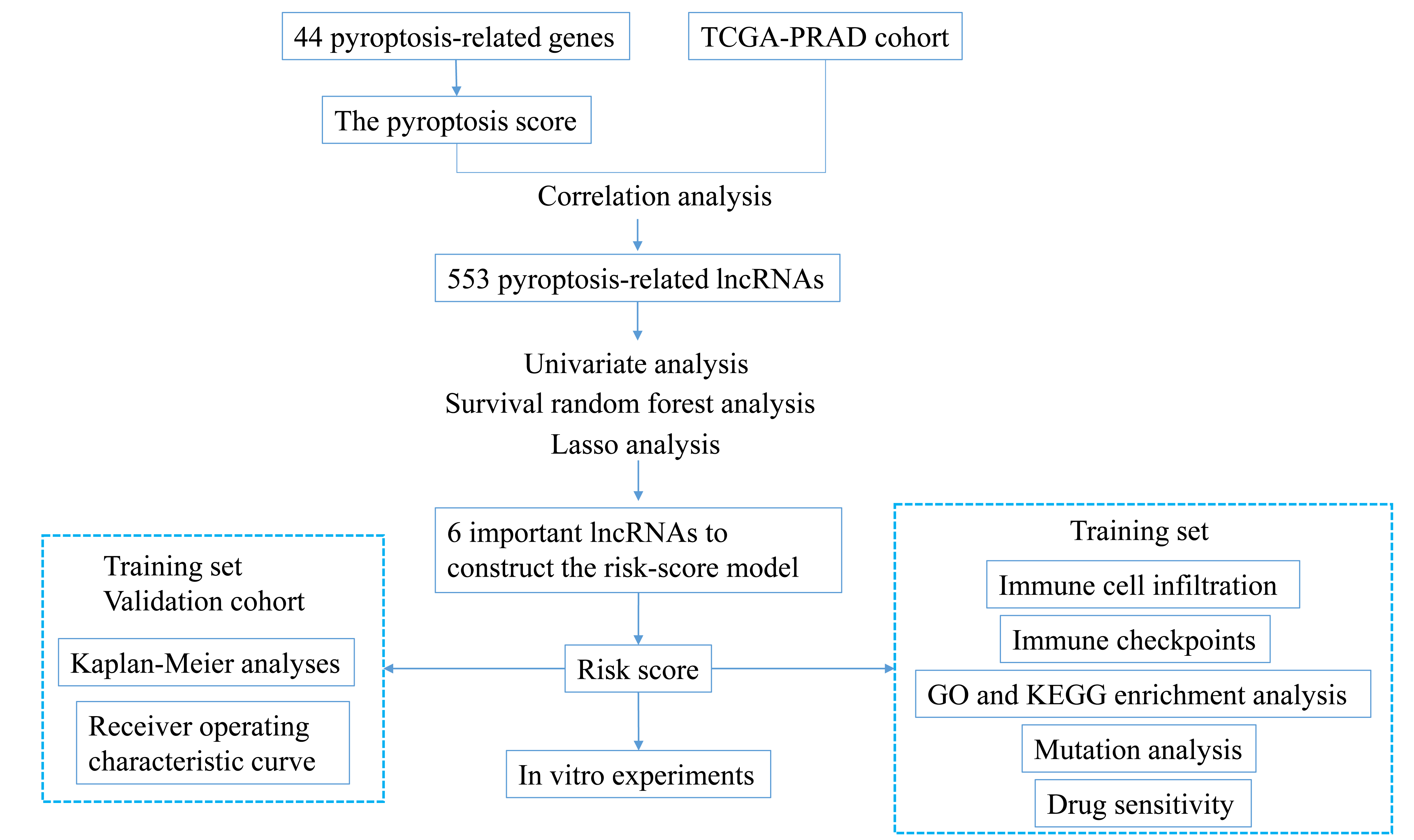

Supplement: Supplementary Figure S1 [file Image_1.jpg]

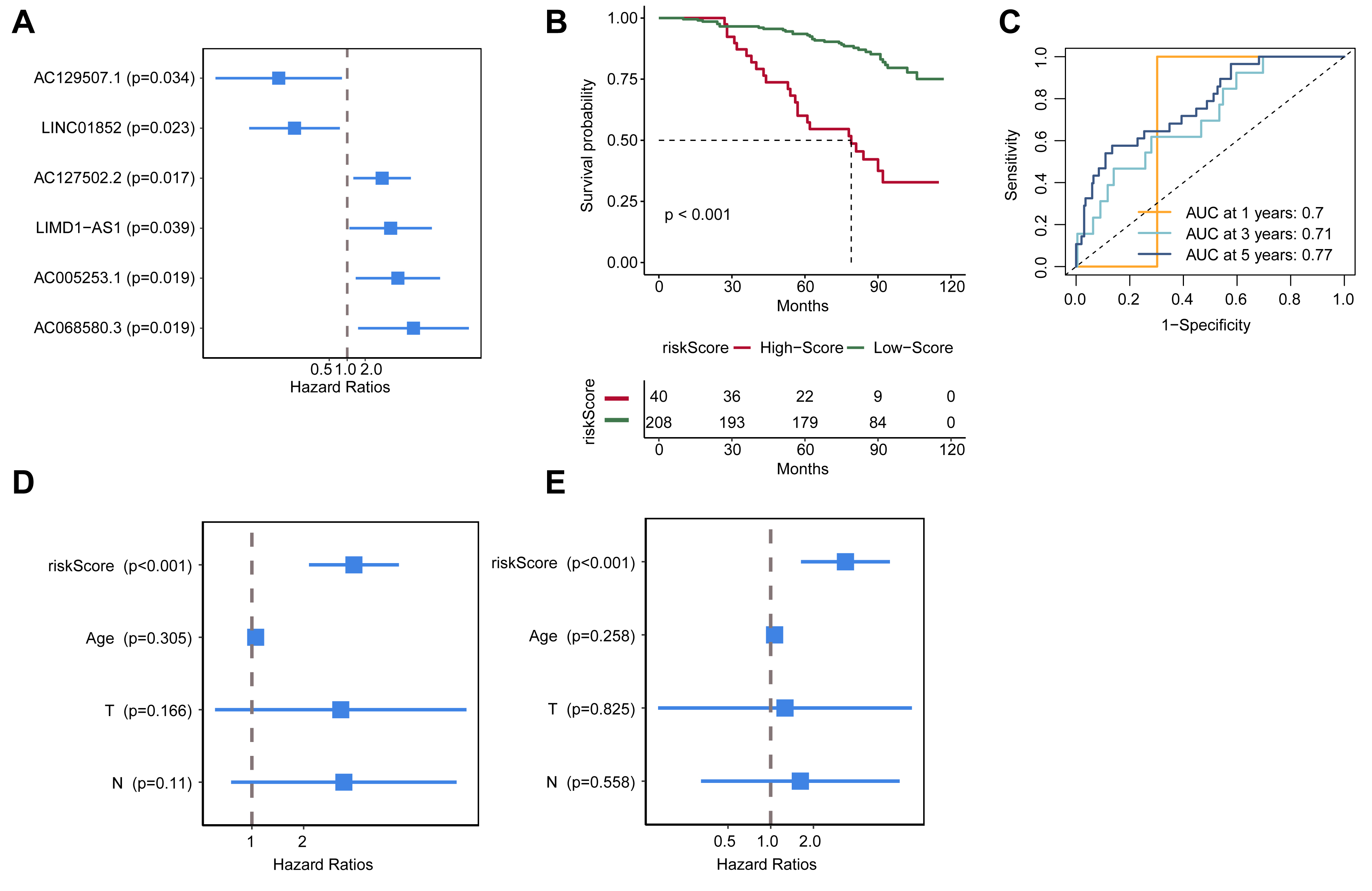

Supplement: Supplementary Figure S2 [file Image_2.jpg]

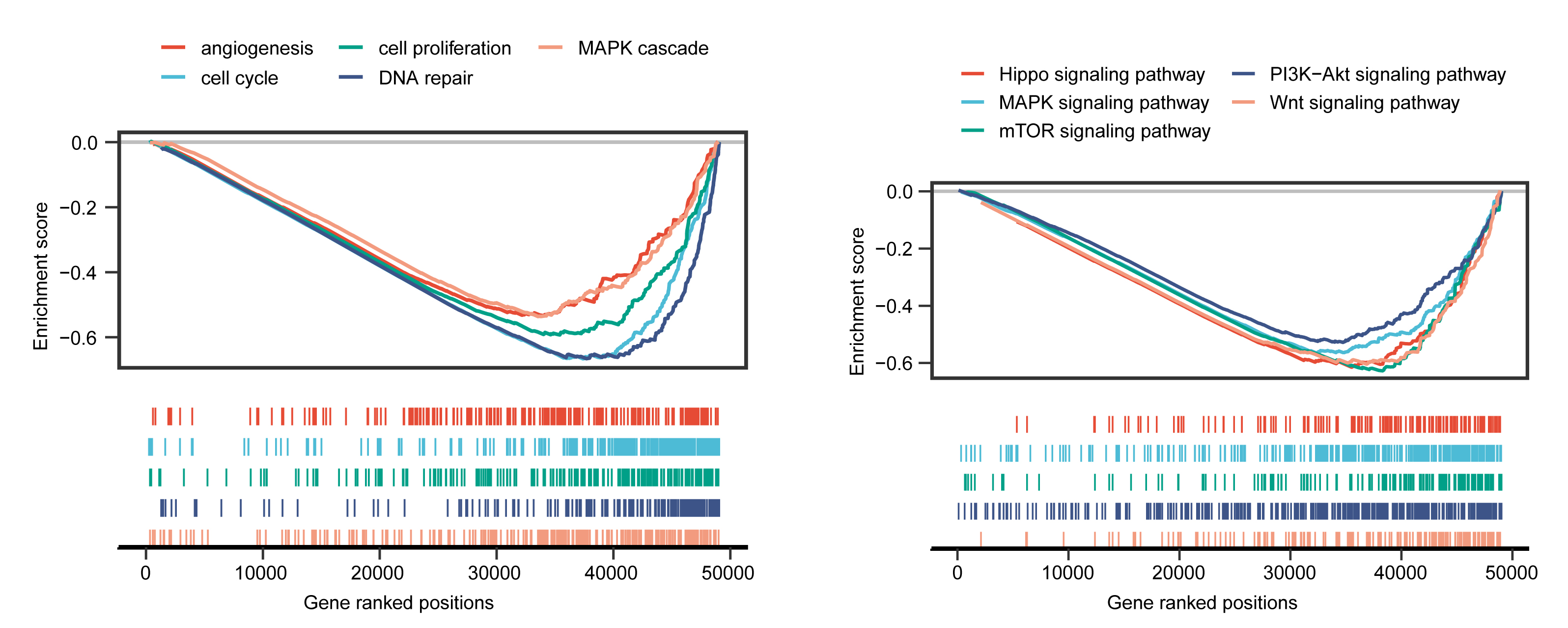

Supplement: Supplementary Figure S3 [file Image_3.jpg]
